# Ophthalmic *Thelazia callipaeda* infections: first feline and new canine imported cases in Germany

**DOI:** 10.1007/s00436-020-06785-2

**Published:** 2020-07-06

**Authors:** Silva L. M. R., S. Spoerel, L. Wiesner, M. Klein, N. Pantchev, A. Taubert, C. Hermosilla

**Affiliations:** 1 Institute of Parasitology, Biomedical Research Center Seltersberg, Justus Liebig University Giessen, Schubertstr. 81, 35392 Giessen, Germany; 2 Tierarztpraxis Dr. Kindler, Saarbrücker Allee 7, 65201 Wiesbaden, Germany; 3 Tierarztpraxis Dr. Klein, Bahnhofstr. 30, 67251 Freinsheim, Germany; 4 IDEXX Laboratories, Mörikestr. 28/3, 71636 Ludwigsburg, Germany

**Keywords:** *Thelazia callipaeda*, Thelaziosis, Eye worm, Cat, Dog, Zoonosis

## Abstract

The first case of feline ocular *Thelazia callipaeda* infection and two new canine imported infections in West Germany are here described. The three animals had a history of recent travel to/from other countries. The young adult cat imported from Spain presented an intermittent unilateral ocular discharge. During in-depth ophthalmic examination, a single alive nematode was removed from the conjunctival compartment of the affected eye. Referring to the canine cases, an adult female dog originated from Kenya presented epiphora and mucous whitish-grey discharge of the right eye. During flushing of the nasolacrimal duct two small, thin and long nematodes were removed. Furthermore, a male Borzoi racing dog with regular visit to racing tracks in different countries presented ocular mucous discharge. At ophthalmologic examination, two transparent-whitish vital nematodes were removed. All nematode specimens of the three cases were morphologically identified as adult *T. callipaeda* parasites. The animals were treated orally with milbemycin oxime (2.0 mg/kg; cat) or milbemycin oxime/praziquantel (0.5 mg/kg and 5.0 mg/kg; dogs) twice with 1-week interval resulting in complete resolution of symptoms. The repeated introduction of patent *T. callipaeda-*infected animals, especially from southern and eastern endemic countries, will ease the establishment of ophthalmic thelaziosis in Northern Europe. The male fruit fly, *Phortica variegata*, an intermediate host of *T. callipaeda*, is endemic within European countries. Considering the clinical and zoonotic relevance of ophthalmic thelaziosis, enhanced disease awareness of European medical and veterinarian doctors and in-depth eye examination for proper detection of *T. callipaeda* are crucial for appropriate anthelmintic treatments and to limit spreading of the infection.

## Introduction

*Thelazia* spp. (Spirurida, Thelaziidae) parasites are ophthalmic vector-borne nematodes commonly found in Southern European countries, North America and Asia. In Europe, eye worms have been reported in cattle (*T. rhodesii*, *T. skrjabini*, *T. gulosa*), horses (*T. lacrymalis*) and carnivores (*T. callipaeda*), such as dogs, cats, foxes and wolves. Recently, various reports brought to light *T. callipaeda* infections of dogs but also of cats, rabbits and humans, mainly from poor socio-economic settings in different Asian countries (Otranto and Traversa 2005; do Vale et al. 2019) but also in several European regions. Consequently, this vector-borne helminthiasis is currently considered as an emergent disease (Colella et al. 2016a, 2020).

Transmission of *T. callipaeda* occurs via males of *Phortica variegata* (Drosophilidae, Steganinae) flies. These non-biting dipteran insects (Otranto et al. 2004) uptake first stage larvae (L1) present in the conjunctival fluid. Within 4 weeks, L1 develops into infective L3 in the insect intermediate host and can be transmitted to final hosts during male fly feeding on conjunctival fluids of other permissive hosts (Otranto et al. 2004). Prepatency lasts from 2 to 6 weeks. Adult eye worms can persist in the conjunctival sac and under the third eyelid of infected hosts for more than 1 year (Motta et al. 2014).

Larvae and adult parasites both are involved in the pathogenesis of ocular thelaziosis (Maia et al. 2016). Clinical signs range from asymptomatic/subclinical to moderate or severe ocular disorders, such as epiphora, blepharitis, photophobia, conjunctivitis, keratitis, corneal opacity or ulcers (Otranto and Traversa 2005). Feline infections are less common than canine ones. Thus, feline thelaziosis seems more sporadic, most probably due to intensive cleaning habits of cats, resulting in less availability of L1 to the vectors (Motta et al. 2014) or even in the mechanical removal of adult eye worms.

Parasites within the conjunctival or nasolacrimal duct cause epiphora, ocular discharge, mild to severe conjunctivitis, follicular hypertrophy of the conjunctiva, foreign body sensation, pruritus, conjunctival hyperaemia, swelling, hypersensitivity to light and keratitis (Hermosilla et al. 2004).

Human thelaziosis has been described not only in adults but also in children, including a 6-month-old child (Otranto et al. 2013). Consistently, also in Germany, the first case of human thelaziosis was reported (Dolff et al. 2020). The patient lived in an urban area of Germany and had a history of several flies being detected in the conjunctival sac after cycling tours in Eastern Europe in summer 2018, constituting most probably an imported case. She presented foreign body sensation, epiphora and redness of the left eye, which already lasted for 2 months before discovering a transparent filamentous structure in the left eye. At ophthalmologic examination, four *T. callipaeda* parasites were extracted, and another nematode was removed the next day under local anaesthesia (Dolff et al. 2020).

Here, we report the first case of feline ocular thelaziosis and new canine imported cases in Germany, bringing awareness not only to veterinary but also to medical doctors to this emergent and neglected zoonosis.

## Cases presentation

### Feline case

**Fig. 1:**
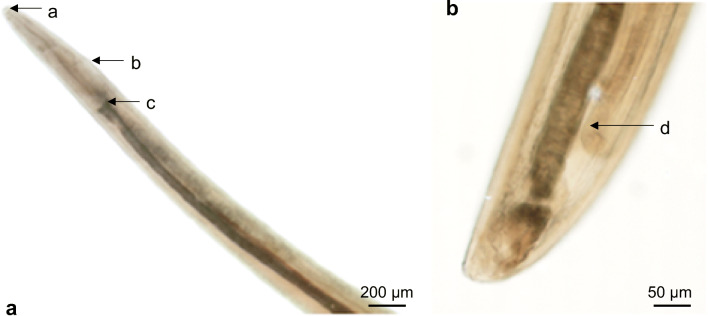
Adult female *Thelazia callipaeda*. **A** Anterior end, crown-shaped buccal capsule (a), vulvar flap (b) anterior to the oesophageal-intestinal junction (c). **B** Posterior end of the female with oocytes (d)

A cat of approximately 1 year of age was adopted from the streets in Southern Spain and thereafter imported to West Germany (Freinsheim, Rhineland-Palatinate), in spring 2015. A few weeks later, during a routine visit at the small animal clinics for a regular health check-up, the cat presented an intermittent unilateral ocular discharge. The cat had shown slight epiphora in the previous days. At physical examination, only epiphora was observed. However, due to the origin of the cat (Spain), an exhaustive in-depth eye examination was performed, and a single vital nematode was collected from the conjunctival compartment of the affected eye and conserved in 70% alcohol for subsequent morphological identification. No nematodes/larvae were observed in the other eye. An adult female *T. callipaeda* parasite (Fig. 1) was morphologically identified by typical morphological characteristics: a yellowish-white nematode of 1.1 cm length with a serrated cuticle, crown-shaped buccal capsule (Fig. 1A, (a)) and vulvar flap (Fig. 1A, (b)) located anterior to the oesophageal-intestinal junction (Fig. 1A, (c)). Moreover, in the uterus in the posterior end of the parasite (Fig. 1B), oocytes (Fig. 1B, (d)) were present. The cat was treated twice with milbemycin oxime (2.0 mg/kg, per os, Interceptor®) with 7-day interval. One month later, no further parasites were observed at ocular control examination.

### Canine case no. 1

At the beginning of 2019, a mixed-Dobermann female spayed dog born in 2008 presented epiphora and mucous whitish-grey discharge in the right eye, which had started several months before clinical submission. The dog showed these ocular signs since being rescued from an animal shelter in Germany (Wiesbaden, Hesse), but it was originally from Kenya. Previous treatments with gentamicin eye drops (TID), followed by ofloxacin eye ointment (TID) were discontinued due to a lack of efficacy. Bacteriological investigations as well as a fluorescein test had shown negative results. At current clinical presentation (Wiesbaden), ophthalmological examination revealed slightly hyperaemic conjunctiva in both eyes, diffuse follicles in the lower conjunctival fornix and a reduced tear film break-up time of 4–5 s. In the right eye, mucous discharge in the nasal corner of the eye was observed and after nasolacrimal flushing, small, thin, long nematodes appeared from the lower lacrimal punctum. A total of two parasites were collected and stored in 70% alcohol for further morphological identification. As treatment, milbemycin oxime and praziquantel (0.5 mg/kg and 5 mg/kg, respectively; per os; Milbemax™) were administrated twice, with 7-day interval. The parasites were identified as *T. callipaeda* by morphological and morphometric features, as previously detailed. At the beginning of March 2019, at control ophthalmologic examination, neither clinical signs nor parasites were observed.

### Canine case no. 2

In January 2020, a 4-year-old, unspayed male Borzoi racing dog from Wiesbaden (Hesse) presented mucous ocular discharge for more than 1 week and was treated with gentamicin eye ointment (TID), without further clinical improvement. The dog was in close contact with several dogs from different countries at various racing tracks, in the Netherlands and Austria. At ophthalmologic examination, the left eye showed no pathological alterations, but the right eye showed a mucopurulent discharge, hyperaemic conjunctiva and follicles. Two transparent-whitish vital nematodes were removed from behind the nictitating membrane and preserved in 70% alcohol for further identification. After careful removal, minor conjunctival haemorrhage on the bulbar side of the nictitating membrane was observed. Nasolacrimal flashing revealed no further nematodes. The parasites were identified as *T. callipaeda* specimen by morphological/morphometric characteristics, as described above. Four days later, the dog showed no alterations of the left eye at control examination, and the right eye presented only mild congestion of the bulbar side of the nictitating membrane. Gentamicin eye ointment (TID) was continued for six more days. For treatment of ophthalmic *T. callipaeda* infection, milbemycin/praziquantel (0.5 mg/kg and 5 mg/kg, respectively; per os; Milbemax™) was administrated twice, with 7-day interval.

## Discussion

Ocular thelaziosis is spreading in Europe (do Vale et al. 2019; Marino et al. 2020), and the number of reports on *T*. *callipaeda* infections has increased throughout the continent (do Vale et al. 2019), not only in domestic and wild animals but also in humans (e.g. Dolff et al. 2020). Reported clinical cases resulted not only from imported but also autochthonous infections, thereby emphasizing the medical importance of this zoonotic disease.

Animal movements between regions, countries and continents bring the risk of relocating pathogens with zoonotic potential that these animals might harbour (Fooks and Johnson 2015). Domestic pet displacements (e.g. import/export, adoption/rescue and travel) from countries that might be endemic for ocular thelaziosis (Magnis et al. 2010; Graham-Brown et al. 2017) are very common in Germany. Dogs and cats from endemic areas, such as Italy, Portugal, France, Greece and Spain, among others, can pose an increased risk of ocular thelaziosis establishment across Europe. Additionally, German owners regularly travel with their pets to neighbouring European countries and other endemic geographic areas. Furthermore, the existence of a sylvatic life cycle for *T. callipaeda* with infections of a large number of wildlife species (i.e. foxes, wolves, beech martens, wild cats, hares and rabbits), could play an important role as natural reservoirs of this eye worm species (Otranto et al. 2013). This constitutes another risk for translocation and emergence of ocular thelaziosis mainly based on migration of *T. callipaeda*-infected wild carnivores which might also spread the disease into previously non-endemic areas of Europe (Otranto et al. 2013). All together, these aspects can explain the geographical expansion of ocular thelaziosis over the last years (Hodžić et al. 2019), which represents a warning signal also for human populations (Otranto et al. 2013).

The male fruit fly *P. variegata* is the obligate intermediate host of *T. callipaeda*, and it is considered as endemic in Europe, including the north of the Alps. Establishment of this particular intermediate host in new geographic areas occurs especially in regions with decaying fruits and fruit plantations, such as strawberry fields and orchards comprising apple and plum trees (Magnis et al. 2010; do Vale et al. 2019) contributing to an increase of ocular thelaziosis episodes. Interestingly, in contrast to a high degree of genetic variability among isolates from Asia (Otranto et al. 2005b), no genetic variation was recorded between individual nematodes collected from different host species (i.e. dogs, cats and foxes) and locations of Europe (i.e. Italy, Germany and the Netherlands). These findings suggest a strict link of *T. callipaeda* to *P. variegata* and a rather low degree of specificity for definitive hosts (Otranto et al. 2013). Ocular thelaziosis was considered in the past as a seasonal disease closely associated with the presence, density and activity of *P. variegata*, which presents high biological activities at temperature ranges of 20–25 °C and at a relative humidity of 50–75% (Otranto et al. 2006a). However, if *T. callipaeda-*infected *P. variegata* males have the ability to overwinter, as already experimentally demonstrated (Otranto et al. 2005a), it may prove to be an important feature to the establishment in cooler northern latitudes and thus autochthonous transmission of *T. callipaeda*. Moreover, the seasonality and crepuscular activities of *P. variegata* males overlap the activity of potentially infected wild species hosts (Otranto et al. 2013), thereby contributing to sylvatic life cycle maintenance.

Canine thelaziosis has been sporadically reported in Germany (Hermosilla et al. 2004; Magnis et al. 2010; Hammerich et al. 2020), but, so far, no case of feline thelaziosis had been reported. On the contrary, neighbouring countries, such as France (Dorchies et al. 2007), Switzerland (Motta et al. 2014) and, more recently, Austria (Hodžić et al. 2019) reported both imported and autochthonous feline ocular thelaziosis. In line with other European reports (Rodrigues et al. 2012), the current feline case was diagnosed in summer, i.e. at times of maximum activity of the vector (Otranto et al. 2006b). However, the fact that the cat originated from Spain, where thelaziosis is endemic (Marino et al. 2020), implies that this case might rather be considered as imported than autochthonous infection. Current canine cases were both diagnosed in January (2019 and 2020), which may either rely on the developmental period of the parasite to reach adult stage (Hodžić et al. 2019), on the capability of adult *T. callip*aeda to survive more than 1 year in the conjunctival fornix of the hosts (Motta et al. 2014) or on the absence of seasonality of *P. variegata* in endemic areas (Maia et al. 2016; do Vale et al. 2019), confirming the distribution of the parasite would be expected to coincide with that of suitable vectors (Otranto et al. 2005b; Dorchies et al. 2007). Referring to previous canine cases in Germany, one was considered an imported case based on a long stay in Italy (Hermosilla et al. 2004), whilst another case seemed autochthonous (Magnis et al. 2010). In the three cases reported here, all animals had regular outdoor access thereby being at risk of infection by potential contact to *T. callipaeda-*infected fruit flies (Rodrigues et al. 2012). Also, pets that coexist with other animals (e.g. sheep, rabbits, cattle, poultry) could be at higher risk of ocular thelaziosis, due to attraction of *P. variegata* to places with large number of hosts (Maia et al. 2016).

Besides *T. callipaeda*, other nematode parasites have been described to cause canine [e.g. *Onchocerca* spp., *Angiostrongylus vasorum* and *Dirofilaria repens* (Hermosilla et al. 2005a, b; Colella et al. 2016b)], feline [e. g. *Onchocerca lupi* (Labelle et al. 2011) and *Gurltia paralysans* (Udiz-Rodríguez et al. 2018)] and human (*O. lupi* and *D. repens*) ocular infections. Hence, in case of conjunctivitis, besides viral or bacterial aetiology, differential diagnosis should include parasitic agents as well (Magnis et al. 2010).

Probably due to the low number of parasites per eye, cats generally show minor to no clinical signs (Motta et al. 2014; Maia et al. 2016), which are easily disregarded by owners and veterinarians (Motta et al. 2014). Intensive cleaning of eye discharges and the small body index of cats were considered as important factors of low feline thelaziosis prevalence (Motta et al. 2014). In addition, reliable in-depth ophthalmic examinations might be less common in cats than in dogs, due to an anticipated increased difficulty, most likely resulting in an underestimation of feline *Thelazia* infections (Motta et al. 2014).

*T. callipaeda* treatments not only clear infections but also avoid the spread of the parasite in previously non-endemic areas (Motta et al. 2012) which is also of major importance regarding the zoonotic potential of the parasite. Treatment of ocular thelaziosis is based on both, mechanical removal of parasites from affected eyes and topical, systemic or spot-on administration of antiparasitic drugs (Rodrigues et al. 2012; Hodžić et al. 2019). Of note, several anthelmintic drugs are ineffective against *T. callipaeda* (i.e. benzimidazoles, quinolines, benzoles, depsipeptides, pyrimidines). Thus, macrocyclic lactones are regarded currently as the drugs of choice to treat *T. callipaeda* infections (Ferroglio et al. 2008; Motta et al. 2012, 2014). High therapeutic efficacies in naturally *T. callipaeda-*infected dogs and cats were reported for different formulations, such as oral (milbemycin oxime/praziquantel) and spot-on (milbemycin oxime/afoxolaner, moxidectin/imidacloprid) application (Motta et al. 2012; Lebon et al. 2019; Otranto et al. 2016, 2019). Two consecutive (7-day interval) milbemycin oxime/praziquantel treatments showed an efficacy of 90.9% and 73.3% in dogs and cats, respectively (Motta et al. 2012), whereas in another study, this drug showed an efficacy of 92.79% in dogs at day 14 after first administration and of 100% at days 28 and 35 (Otranto et al. 2016). Following the second treatment with milbemycin oxime 1 week after the first administration, an accurate concentration of the product in the conjunctiva justifies the higher efficacy against the parasite (Ferroglio et al. 2008). However, a lower efficacy was observed when the treatment was administered without food (Motta et al. 2012). A single treatment by spot-on formulation containing moxidectin 1.0% (+ imidacloprid 10%) resulted in 100% efficacy in naturally infected cats 28 days after treatment (Otranto et al. 2019). In line, an efficacy of 100% was reported in dogs treated with a single spot-on application of 2.5% moxidectin and 10% imidacloprid (Otranto et al. 2016).

Prophylaxis of ocular thelaziosis can be achieved by monthly administration of macrocyclic lactones (Ferroglio et al. 2008; Motta et al. 2012; Otranto et al. 2016, 2019; Lebon et al. 2019) resulting in prevalence reduction and therefore limited human infection risk in endemic areas (Otranto et al. 2013). Moreover, an in-depth eye inspection, even in the absence of ocular manifestations, should be included in routine clinical examinations of dogs and cats throughout the year (Maia et al. 2016). This recommendation not only refers to endemic areas but to all European countries where ocular thelaziosis was ever reported. Actually, if established deworming protocols (e.g. see ESCCAP.org) would be followed in a similar fashion as recommended for dirofilariosis and other helminth control, animals living in *T. callipaeda* endemic areas would benefit of an early elimination of larval stages, thus potentially abrogating host-parasite transmission chain (Motta et al. 2012, 2014). In this context, it seems necessary to keep in mind that older animals are at higher risk of infection due to the decrease in deworming frequency (deworming is usually associated with vaccinations and other veterinary actions, more frequent during the first years of animals’ life). Moreover, owners tend to consider anthelmintic treatments less necessary in older animals, resulting in a high number of adult pets remaining untreated (Motta et al. 2014).

Considering the fast spread of ocular thelaziosis within European countries and the zoonotic relevance of this parasitosis, increased awareness of medical and veterinarian surgeons seems important to limit the risk of further infections, especially in Germany where awareness is low even though a human case was recently reported (Dolff et al. 2020).
